# Predictors of Persistence of Anxiety, Hyperarousal Stress, and Resilience During the COVID-19 Epidemic: A National Study in Iran

**DOI:** 10.3389/fpsyg.2021.671124

**Published:** 2021-09-29

**Authors:** Hamid Sharif Nia, Elham Akhlaghi, Samaneh Torkian, Vahid Khosravi, Reza Etesami, Erika Sivarajan Froelicher, Saeed Pahlevan Sharif

**Affiliations:** ^1^Department of Nursing, Mazandaran University of Medical Science, Sari, Iran; ^2^Department of Medical Surgical Nursing, School of Nursing and Midwifery, Iran University of Medical Sciences, Tehran, Iran; ^3^Department of Epidemiology, School of Public Health, Iran University of Medical Sciences, Tehran, Iran; ^4^Health Education and Promotion, School of Public Health, Shahid Sadoughi University of Medical Sciences, Yazd, Iran; ^5^Department of Statistics, Shahid Bahonar University, Kerman, Iran; ^6^Department of Physiological Nursing, Schools of Nursing, University of California, San Francisco, San Francisco, CA, United States; ^7^Department of Epidemiology & Biostatistics, Schools of Medicine, University of California, San Francisco, San Francisco, CA, United States; ^8^Taylor's Business School, Taylor's University, Subang Jaya, Malaysia

**Keywords:** anxiety, COVID-19, mental health, hyperarousal stress, resilience

## Abstract

**Background:** The coronavirus pandemic can cause unprecedented global anxiety, and, in contrast, resilience can help the mental health of people in stressful situations. This study aimed to assess anxiety, hyperarousal stress, the resilience of the Iranian population, and their related factors during the coronavirus disease 2019 (COVID-19) epidemic.

**Methods:** A cross-sectional study was conducted in 31 provinces in Iran between March 18 and 25, 2020. A four-part questionnaire, including the demographic information, the State-Trait Anxiety Inventory (STAI-y1—a 20-item standard questionnaire for obvious anxiety), the Connor–Davidson Resilience Scale (CD-RISC—a 25 item standard questionnaire), and the stress hyperarousal subscale from the Impact of Event Scale-Revised (IES-R), was used to collect data. The ordinal multivariable generalized estimating equation (GEE) model was used to identify correlates of the psychological factors mentioned above. The Fisher exact test was used to investigate the relationship between anxiety, stress, resilience, and the COVID-19 outbreak. All analyses were conducted with SPSS 26 and GIS 10.71.

**Results:** The findings show that most people had moderate-to-severe anxiety (80.17%) and a high level of resilience (96.4%) during the COVID-19 epidemic. The majority of participants had a moderate level of stress (58.9%). The lowest and highest prevalences of psychiatric disorders were in Sistan and Baluchestan (3.14 cases per 100,000 people) and Semnan (75.9 cases per 100,000 people) provinces, respectively. Men and unmarried people were the only variables significantly associated with anxiety and resilience. Age, gender, and education were significantly associated with hyperarousal stress.

**Conclusion:** The high and moderate levels of anxiety and stress in Iranians can have negative effects on the well-being and performance of the people and can lead to serious problems. Also, high resilience during negative life events (such as the COVID-19 pandemic) is associated with the well-being in the lives of people. The results of this study can be used in interventions and other psychological studies.

## Introduction

The novel coronavirus disease 2019 (COVID-19) first appeared in Wuhan, Hubei Province, China, in late 2019, and it rapidly spread throughout China and to nearly every country in the world (Bogoch et al., 2020). A pandemic was declared by World Health Organization (WHO) in March 2020 (Zhu et al., 2020). According to the WHO statistics, more than 184,820,132 people have been infected, and more than 4,002,209 deaths have been recorded worldwide as of July 8, 2021. Iran has the 13th highest number of coronavirus infection cases in the world. Between February 19 and 23, 2020, Iran reported 43 confirmed cases and 8 deaths in Qom. Since July 2021, the coronavirus toll in Iran reached 3,327,526 infections and 85,397 deaths (World Health Organization, 2021). The coronavirus outbreak reached its peak in 2 months in China and in <1 month in Iran (World Health Organization, 2021).

Because of the high transmissibility of the COVID-19, it can spread from person to person even if the person is asymptomatic (Li Q. et al., 2020; Pan et al., 2020). The very high number of confirmed cases and high fatality rates have resulted in psychological problems such as stress, anxiety, and depression among the medical staff as well as in the community (Kang et al., 2020; Qiu et al., 2020; Xiang et al., 2020). The general panic caused by the coronavirus outbreak has increased the symptoms of anxiety (Huang and Zhao, 2020). These symptoms are related to the natural protective response of the body against the stress of the pandemic (Maunder et al., 2003). The stress response system has both positive and negative aspects (Nesse et al., 2016); while the stress response system causes symptoms, it also has long-term benefits by increasing adaptability; therefore, responding to stress is to some extent a necessary and beneficial mechanism (Charney, 2004).

Another response to stress is the activation of the sympathetic system coupled with symptoms such as increased arousal, fever, sweating, and respiratory rate (Nesse et al., 2016). To that end, research has shown that anxiety can also cause dyspnea (Hinz et al., 2012; Holas et al., 2017). For some people, it can be confusing to identify the difference between symptoms of stress and coronavirus because coronavirus shares some of the symptoms with panic, such as fever, sweating, and dyspnea (Chen et al., 2020; Huang et al., 2020). Thus, excessive and constant anxiety is a common and debilitating problem that causes considerable suffering for the individual and their loved ones and is expensive due to the overuse of health services (Fink et al., 2010).

Anxiety, as a form of psychological stress, can also cause physiological changes and weakens the immune system (Liu et al., 2020). The immune system can protect against pathogens and can have positive effects by reducing stress and anxiety of patients (Reed and Raison, 2016; Li G. et al., 2020).

One study found that symptoms of anxiety in the COVID-19 epidemic were present among people under the age of 35 and those who spent a lot of time focusing on the epidemic and did not show a difference in anxiety between men and women (Huang and Zhao, 2020), while women were more anxious than men in other studies during this pandemic (Guo et al., 2016; Gao et al., 2020).

The field of psychology recognized the interaction between the individual and the environment (Masten and Reed, 2002) in the late nineteenth and early twentieth centuries. Connor and Davidson regard resilience as the ability of an individual to maintain a psychological balance in perilous situations (Connor and Davidson, 2003).

Considerable research on the role of resilience under various situations has shown that resilience can help people in the face of stressful life adversity (Izadinia et al., 2010). It can also modulate levels of stress and disability in stressful situations and enhance problem-solving skills (Pinquart, 2008). Resilient people use coping skills to deal with stress (Campbell-Sills et al., 2006). Resilience is about improving social activities and overcoming problems despite exposure to severe stress, anxiety, and difficult life experiences. Resilience is the ability to grow, mature, and increase the capacity of an individual against adverse conditions (Amiry, 2019). Resilience is an adaptation that manifests itself during debilitating problems and stresses. This definition of resilience states that there is a complex interaction between a dangerous situation and the protective factors (Cénat and Derivois, 2014).

It is also important to prevent anxiety among people, to teach them health principles, and to maintain calmness (Farnoosh et al., 2020). Findings from this study can guide the designing and implementation of policies for mental health interventions to effectively address this challenge. Based on the limited evidence of the stress during earlier epidemics, this study hypothesized that, given the severity of the COVID-19 pandemic, similar adverse psychological responses may manifest (Maunder et al., 2003). The main purpose of this study is to measure the severity of anxiety, stress, and resilience in Iranians in order to determine the current mental health needs and to design interventions for the Iranian population.

## Methods

A cross-sectional study was used to evaluate the psychological responses in the general population in Iran during the COVID-19 pandemic from March 18 to 25, 2020. Data were collected with a web-based questionnaire in 31 provinces in Iran using a snowball-sampling technique. The aim was to measure anxiety, hyperarousal stress, and resilience in this critical situation. A total of 70,180 persons completed the questionnaire. This study was limited to individuals who had access to the web (to complete the questionnaire) and were literate. Participation in this study was voluntary and confidential.

A four-part questionnaire, including the demographic information, the State-Trait Anxiety Inventory (STAI-y1—a 20-item standard questionnaire for obvious anxiety), the Connor–Davidson Resilience Scale (CD-RISC—a 25-item standard questionnaire), and the stress hyperarousal subscale from the Impact of Event Scale-Revised (IES-R), was used to collect the data.

*Demographic variables* included gender (male and female), age (<30, 31–40, 41–50, and >50), marital status (married, single, divorced, and widowed), chronic pre-existing conditions (yes or no), education (diploma or less, associate degree, bachelor, masters, and doctorate), job (health workers and others), and economic status (good, moderate, and poor).

*The anxiety measure* STAI-y_1_ has 20 items, and all items were rated on a 5-point scale (from “Almost Never = 1” to “Almost Always=4”). A score of four indicates greater anxiety, but for questions 1, 2, 5, 8, 10, 11, 15, 16, and 19, a high score indicates a lack of anxiety, and grading weights for these questions are reversed (Julian, 2011). This questionnaire was used to evaluate the anxiety symptoms during the past week. The STAI-y1 questionnaire was scored from 20 to a maximum of 80 points. STAI-y_1_ scores are commonly classified as “no or low anxiety” (20–37), “moderate anxiety” (38–44), and “high anxiety” (45–80) (Козьминых, 2019).

*The resilience measure* CD-RISC consists of 25 items that are evaluated on a 5-point Likert scale ranging from 0 to 4: not true at all (0), rarely true (1), sometimes true (2), often true (3), and true nearly all of the time (4). These ratings result in a number between 0 and 100, and higher scores indicate a higher resilience (Connor and Davidson, 2003). The cut-point for the resilience questionnaire was based on the Likert score, and the average score of the questionnaire was used (Garland, 1991; Narli, 2010). Accordingly, participants with mean scores of ≤ 1.33, 1.34–2.66, and 2.67–4 were regarded as having low resilience, moderate resilience, and high resilience, respectively.

*The stress hyperarousal subscale* consisted of six questions from the IES-R questionnaire. IES-R included the three subscales: intrusion (eight items), avoidance (eight items), and hyperarousal (six items); we used only the hyperarousal subscale (Beck et al., 2008). The 5-point Likert scale response options were used (0–4): not true at all (0), rarely true (1), sometimes true (2), often true (3), and true nearly all of the time (4). The score ranges are from 0 to 24, and higher scores indicate more stress (Christianson and Marren, 2012). The high reliability and the validity of the three questionnaires have been established in earlier studies (Panaghi and Mogadam, 2006; Jowkar et al., 2010; Keyhani et al., 2015; Mahram, 2018). In this study, Cronbach's alpha for the anxiety questionnaire, the stress questionnaire, and the resilience questionnaire were 0.85, 0.73, and 0.93, respectively.

Also, the data of incidence of COVID-19 were obtained from the cases announced from the latest news of the provinces between March 6 and 20, 2020 to investigate the relationship between the COVID-19 outbreak and anxiety, stress, and resilience.

### Ethical Approval and Consent to Participate

Ethical approval for this study was obtained from the Mazandaran University of Medical Sciences. The Ethical Code IR.MAZUMS.REC.1399.7293 was assigned to this study. On the first page of the questionnaire, the objectives of the study, the email ID for questions, the ethics of the study, and information about the optional participation in the study and their anonymity given to the participants were explained.

### Statistical Procedures

In this study, the dependent variables had three categories; therefore, we used the ordinal multivariable generalized estimating equation (GEE) models to identify correlates of the psychological factors mentioned above. Odds ratios (ORs) with 95% confidence intervals (CI) were reported. The geographic information system (GIS) was used to draw hotspots of anxiety, stress, and resilience. This method used the median, and the hotspots for anxiety, stress hyperarousal, and resilience in Iran were plotted. The cutoffs were the same as those mentioned above, but the median was used instead of the mean. The incidence risk of COVID-19 (confirmed COVID-19 cases/population at risk) between March 6 and 19, 2020 was shown in a bar chart. The Fisher′s exact test was used to investigate the relationship between the COVID-19 outbreak with anxiety, stress, and resilience. A *P* <0.05 was considered statistically significant. We conducted all analyses using SPSS 26 and GIS 10.71.

## Results

In this survey, most of the participants were male (64.3%), were married (75.8%), had a bachelor degree (37.6%), had a medium-income level (70.4%), and had no chronic pre-existing conditions (80.9%). The mean age (±SD) of the participants was 41.21 (±11.71) years.

The prevalence of anxiety, stress, and resilience in subgroups by demographic variables is shown in Table 1. The anxiety, stress hyperarousal, and resilience in Iranians during the COVID-19 epidemic were means (SD) of 47.64 (±11.51), 10.28 (±3.91), and 64.74 (±16.44), respectively. In this study, 59.4% of the people reported high anxiety, 20.8% reported moderate anxiety, and 19.8% reported low anxiety. Most of the Iranians had moderate-to-severe anxiety (80.17%) during the COVID-19 epidemic. A high level of stress hyperarousal was reported by 6.6%; a moderate level was reported by the majority of people (59.4%), and 34% reported a low level of stress. Most of the people reported moderate (47.1%) and high (48.9%) levels of resilience.

**Table 1:** Characteristics of participants according to the demographic and the psychological variables during the COVID-19 pandemic (*n* = 70,180).

**Variables**	***n* (%)**	**Resilience**			**Anxiety**			**Stress**		
		**Low**	**Moderate**	**High**	**Low**	**Moderate**	**High**	**Low**	**Moderate**	**High**
**Gender**										
Male	25,037 (35.7)	902 (3.6)	9,556 (38.2)	14,579 (58.2)	12,063 (48.2)	686 (2.7)	12,288 (49.1)	8,580 (34.3)	14,738 (58.9)	1,719 (6.9)
Female	45,143 (64.3)	1,916 (4.2)	23,491 (52.0)	19,736 (43.7)	1,857 (4.1)	13,877 (30.7)	29,409 (65.1)	15,279 (33.8)	26,968 (59.7)	2,899 (6.4)
Total	70,180	2,818 (4.0)	33,047 (47.1)	34,318 (48.9)	13,920 (19.8)	14,697 (20.8)	41,697 (59.4)	23,856 (34.0)	417.6 (59.4)	4,618 (6.6)
**Age (years)**										
(8–30)	11,568 (16.9)	411 (3.6)	5,467 (47.3)	5,690 (49.2)	2,165 (18.7)	2,648 (22.9)	6,755 (58.4)	3,977 (34.4)	6,826 (59.0)	765 (6.6)
(31–40)	24,513 (35.7)	995 (4.1)	11,609 (47.4)	11,909 (48.6)	4,669 (19.0)	5,324 (21.7)	14,520 (59.2)	8,175 (33.3)	14,666 (59.8)	1,672 (6.8)
(41–50)	17,728 (25.8)	723 (4.1)	8,370 (47.2)	8,635 (48.7)	3,628 (20.5)	3,536 (19.9)	10,564 (59.6)	6,074 (34.3)	10,539 (59.4)	1,115 (6.3)
(51–99)	14,786 (21.6)	614 (4.2)	6,857 (46.4)	7,315 (49.5)	3,190 (21.6)	2,687 (18.2)	8,909 (60.3)	5,114 (34.6)	8,723 (59.0)	949 (6.4)
**Marital status**										
Single	14,097 (20.1)	486 (3.4)	6,163 (43.7)	7,448 (52.8)	2,845 (20.2)	4,487 (31.8)	6,765 (48.0)	4,691 (33.3)	8,419 (59.7)	987 (7.0)
Divorce/Widowed	2,901 (4.1)	135 (4.7)	1,468 (50.6)	1,298 (44.7)	257 (8.9)	814 (28.1)	1,830 (63.1)	972 (33.5)	1,735 (59.8)	194 (6.7)
Married	53,182 (75.8)	2,197 (4.1)	25,416 (47.8)	25,569 (48.1)	10,818 (20.3)	9,262 (17.4)	33,102 (62.2)	18,193 (34.2)	31,552 (59.3)	3,437 (6.5)
**Chronic pre-existing conditions**										
No	56,778 (80.9)	2,286 (4.0)	26,721 (47.1)	27,771 (48.9)	11,142 (19.6)	12,019 (21.2)	33,617 (59.2)	19,240 (33.9)	33,809 (59.5)	3,729 (6.6)
Yes	13,402 (19.1)	532 (4.0)	6,326 (47.2)	6,544 (48.8)	2,778 (20.7)	2,544 (19.0)	8,080 (60.3)	4,616 (34.4)	7,897 (58.9)	889 (6.6)
**Education**										
Diploma and less	18,526 (26.4)	772 (4.2)	8,855 (47.8)	8,899 (48.0)	3,544 (19.1)	3,829 (20.7)	11,153 (60.2)	6,483 (35.0)	10,961 (59.2)	1,082 (5.8)
Associate degree	7,170 (10.2)	311 (4.3)	3,387 (47.2)	3,472 (48.4)	1,487 (20.7)	1,379 (19.2)	4,304 (60.0)	2,355 (32.8)	4,375 (61.01)	440 (6.1)
Bachelor	26,373 (37.6)	1,029 (3.9)	12,459 (47.2)	12,885 (48.9)	4,868 (18.5)	5,693 (21.6)	15,812 (60.0)	8,969 (34.0)	15,612 (59.4)	1,792 (6.8)
Masters Doctorate	18,111 (25.8)	706 (3.9)	8,346 (46.1)	9,059 (50.0)	4,021 (22.2)	3,662 (20.2)	10,428 (57.6)	6,049 (33.4)	10,758 (59.4)	1,304 (7.2)
**Job**										
Other	63,460 (60.4)	2,558 (4.0)	2,558 (4.0)	31,154 (49.1)	12,823 (4.0)	12,959 (20.4)	37,678 (59.4)	21,636 (34.1)	37,618 (59.3)	4,206 (6.6)
Health workers	6,720 (9.6)	260 (3.9)	260 (3.9)	3,161 (47.0)	1,097 (16.3)	1,604 (23.9)	4,019 (59.8)	2,220 (33.0)	4,088 (60.8)	412 (6.1)
**Economic situation**										
Good	11,449 (16.3)	463 (4.0)	5,585 (48.8)	5,401 (47.2)	1,841 (16.1)	2,555 (22.3)	7,053 (61.6)	3,830 (33.5)	6,890 (60.2)	729 (6.4)
Moderate	49,382 (70.4)	1,987 (4.0)	23,186 (47.0)	24,209 (49.0)	9,758 (19.8)	10,266 (20.8)	29,358 (59.5)	16,848 (34.1)	29,237 (59.2)	3,297 (6.7)
Poor	9,348 (13.3)	368 (3.9)	4,275 (45.7)	4,705 (50.3)	2,321 (24.8)	1,742 (18.6)	5,285 (56.5)	3,178 (34.0)	5,578 (59.7)	592 (6.3)

**Figure 1:**
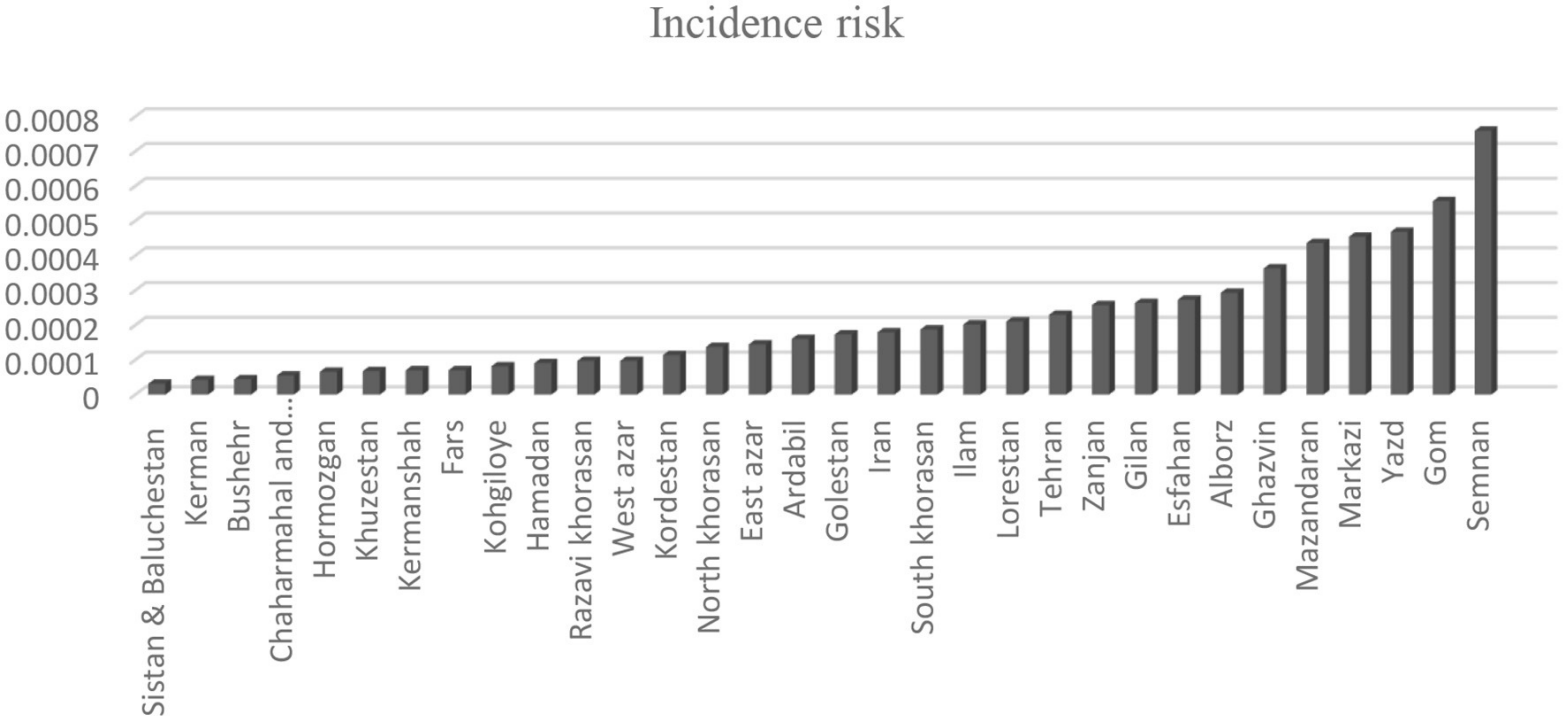
The incidence risk of COVID-19 in the provinces of Iran from March 6 to 28, 2020.

The incidence of COVID-19 in the provinces is shown in Figure 1. The lowest and highest incidence risks of COVID-19 were in Sistan and Baluchestan (3.14 cases per 100,000 people) and in Semnan (75.9 per cases 100,000 people) provinces, respectively.

**Figure 2:**
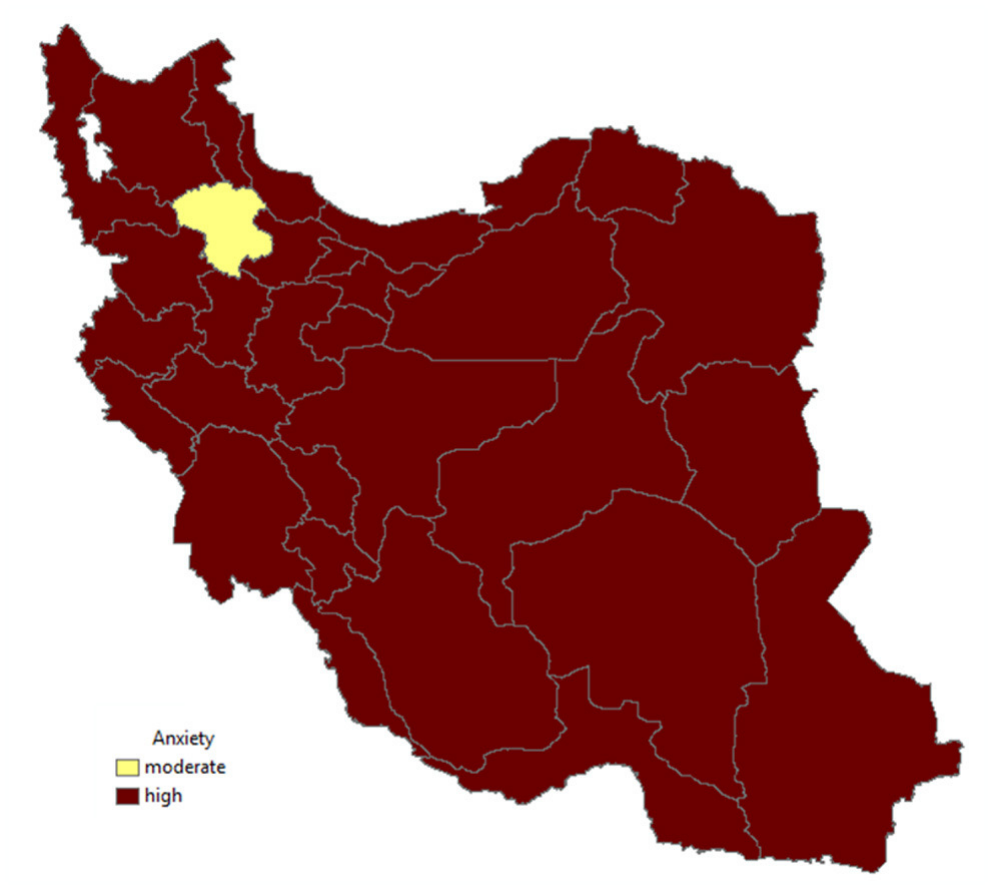
Anxiety in the provinces of Iran.

The median distribution of the anxiety score is shown in Figure 2. As shown in Figure 2, the people in almost all parts of Iran were highly anxious.

**Figure 3:**
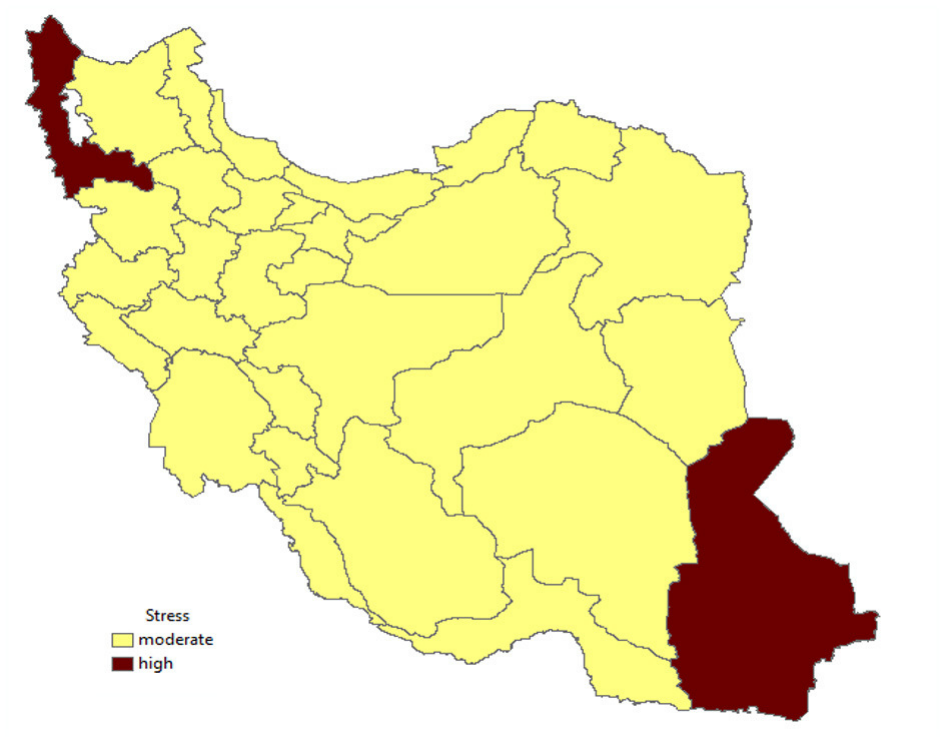
Stress in the provinces of Iran.

Figure 3 shows that many parts of Iran have moderate levels of stress.

**Figure 4:**
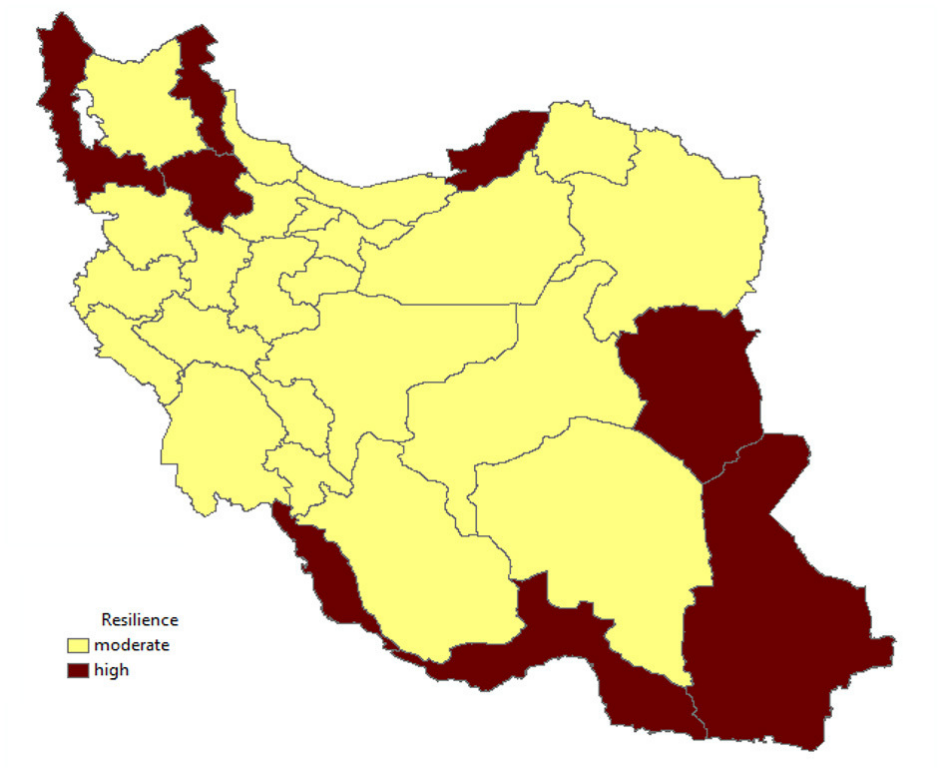
Resilience in the provinces of Iran.

Figure 4 also shows the high and moderate resistances of all parts of Iran.

**Figure 5:**
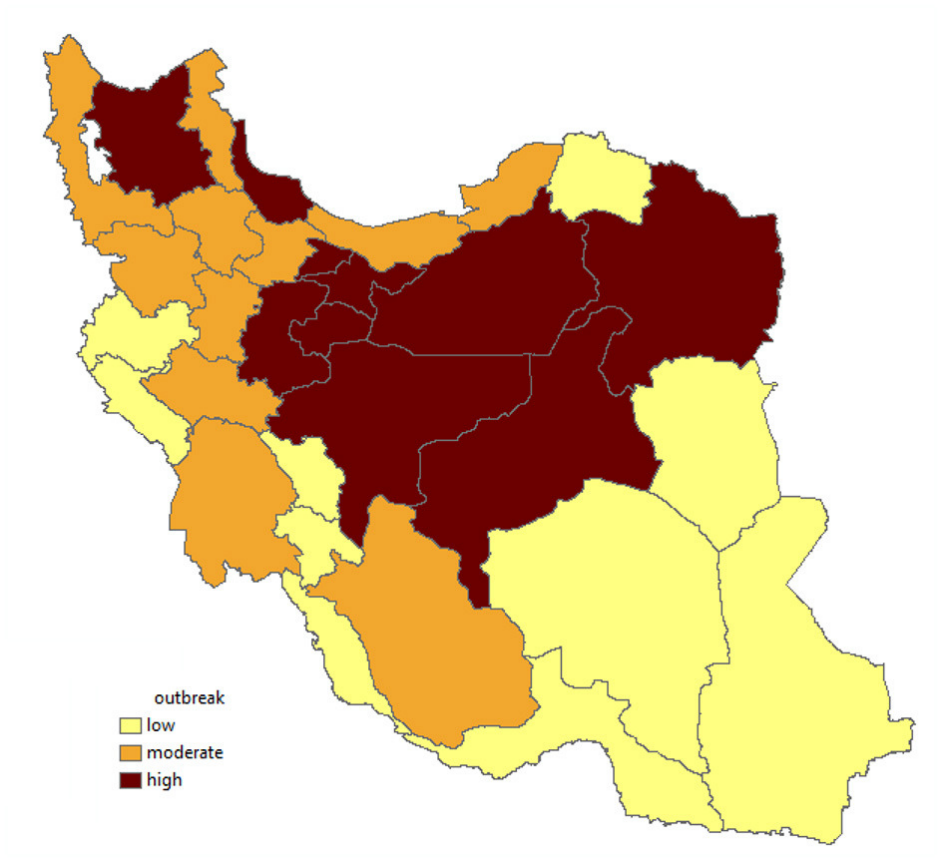
Outbreak of COVID-19 in the provinces of Iran.

The distribution of the incidence cases of COVID-19 in Iran between March 6 and 28, 2020 is shown in Figure 5.

The association between the demographic variables and the psychological impact of the COVID-19 outbreak is shown in Table 2. The following demographic variables, gender (female), age (>50 years), marital status (being married), having a chronic pre-existing condition, education (masters degree), employment (other jobs), and economic status (being poor), were reference groups for the GEE models.

**Table 2:** The ordinal multivariable generalized estimating equation models to determine the correlates of the psychological impact of the COVID-19 in Iran (*n* = 70,180).

**Variables**	**Resilience**	**Anxiety**		**Stress hyperarousal**	
	**OR (95% CI)**	***p*-value**	**OR (95% CI)**	***p*-value**	**OR (95% CI)**	***p*-value**
**Gender**						
Male	1.76 (1.70, 1.82)	** <0.001**	0.28 (0.25, 0.26)	** <0.001**	0.99 (0.96, 1.03)	0.960
Female	1	1	1	1	1	1
**Age (years)**						
(8–30)	0.98 (0.93, 1.03)	0.587	1.03 (0.97, 1.08)	0.243	0.99 (0.94, 1.05)	0.941
(31–40)	0.98 (0.94, 1.02)	0.436	0.96 (0.92, 1.01)	0.130	1.05 (1.00, 1.09)	**0.022**
(41–50)	0.98 (0.94, 1.03)	0.559	0.95 (0.91, 0.99)	**0.047**	1.00 (0.96, 1.04)	0.838
(51–99)	1	1	1	1	1	1
**Marital status**						
Single	1.25 (1.20, 1.30)	** <0.001**	0.57 (0.55, 0.59)	<0.001	1.05 (1.01, 1.09)	**0.014**
Divorced/Widowed	1.02 (1.00, 1.10)	** <0.001**	0.85 (0.79, 0.92)	** <0.001**	1.05 (0.97, 1.13)	0.217
Married	1	1	1	1	1	**1**
**Chronic pre-existing conditions**						
No	1.00 (0.96, 1.04)	0.721	0.97 (0.93, 1.01)	0.269	1.01 (0.97, 1.05)	0.591
Yes	1	1	1	1	1	1
**Education**						
Diploma and less	0.96 (0.92, 1.00)	0.105	1.03 (0.98, 1.08)	0.150	0.91 (0.87, 0.95)	** <0.001**
Associate degree	0.96 (0.91, 1.02)	0.223	1.02 (0.97, 1.08)	0.353	0.99 (0.94, 1.05)	0.833
Bachelor	1.00 (0.91, 1.01)	0.847	1.03 (0.99, 1.08)	0.067	0.97 (0.93, 1.00)	0.125
Masters/doctorate	1	1	1	1	1	1
**Job**						
Health workers	0.96 (0.92, 1.01)	0.214	0.96 (0.91, 1.01)	0.159	1.00 (0.95, 1.05)	0.828
Other	1	1	1	1	1	1
**Economic situation**						
Good	0.98 (0.93, 1.05)	0.695	1.05 (0.99, 1.12)	0.060	1.00 (0.94, 1.06)	0.964
Moderate	1.01 (0.96, 1.06)	0.636	1.02 (0.97, 1.07)	0.335	0.98 (0.94, 1.03)	0.508
Poor	1	1	1	1	1	1

*The bold values are indicate statistical significance*.

Gender and marital status were the only variables significantly associated with anxiety and resilience. Being male were significantly associated with a higher resilience level (OR = 1.76, 95% CI: 1.70, 1.82) and a lower anxiety level (OR = 0.28, 95% CI: 0.25, 0.26). Marital status was significantly associated with the CD-RISC and STAI levels. Being single (OR = 1.25, 95% CI: 1.20, 1.30) and being widowed/divorced were significantly associated (OR = 1.02, 95% CI: 1.00, 1.10) with higher resilience. Also, being single (OR = 0.57, 95% CI: 0.55, 0.59) and divorced/widowed (OR = 0.57, 95% CI: 0.55, 0.59) were significantly associated with lower anxiety.

Other sociodemographic variables including age, underlying chronic disease, education, job, and economic situation were not associated with the CD-RISC and STAI levels. Age groups ≤ 30, being single, diploma, and lower education level were significantly associated with hyperarousal stress. Being single (OR = 1.05, 95% CI: 1.01, 1.09) and age groups (≤ 30 years) (OR = 1.05, 95% CI: 1.00, 1.09) were significantly associated with a higher IES-R subscale level and those who had a diploma or education level (OR = 1.05, 95% CI: 1.00, 1.09) were significantly associated with a lower IES-R subscale level. Other sociodemographic variables including gender, widowed/divorced, age (except age groups ≤ 30), underlying chronic disease, education (except diploma and less education), job, and economic situation were not associated with the IES-R subscale levels.

The median resilience score was significantly associated (*p* = 0.044) with an outbreak, but the median anxiety (*p* = 1.000) and stress (*p* = 0.073) scores had no significant relationship with the COVID-19 outbreak.

## Discussion

The salient findings of this study include the following. Most of the Iranians reported moderate-to-severe levels of anxiety, moderate stress, and resilience during the COVID-19 pandemic. These findings confirm those reported during the initial phase of the COVID-19 outbreak in China, where about one-third of the general population in China reported moderate-to-severe anxiety (Wang et al., 2020). In Rome, 89.4% of students reported an increase in stress (66% moderate and 23.4% high stress), which remained consistent with our results (Quintiliani et al., 2021). The prevalence of anxiety in a systematic review and meta-analysis in 2016 in Iranians showed mild (31%), moderate (37%), intense (19%), and highly intense (2%) levels of anxiety (Valizadeh et al., 2016). These findings suggest that an increase in the prevalence of high anxiety during the COVID-19 epidemic was reported.

Consistent with this study, Limcaoco et al. in their study reported higher levels of anxiety in women during the COVID-19 epidemic (Limcaoco et al., 2020). Consistent with our findings, Wang et al. showed in their study that gender and age were associated with anxiety and that anxiety rates were higher in women and younger people (<40 years). However, in our study, <40 years of age was not associated with anxiety (Wang et al., 2021). A meta-analysis study conducted until May 2020 showed that the prevalence of stress in five studies with a total sample size of 9,074 was 29.6% and the prevalence of anxiety in 17 studies with a sample size of 63,439 was 31.9%. The prevalence of stress in this meta-analysis was higher than that of the severe stress in the present study but the prevalence of anxiety was lower (Salari et al., 2020).

High levels of stress and anxiety were not associated with the COVID-19 epidemic in this study. We guess that stress and anxiety are associated with the two important consequences of the COVID-19 pandemic: availability of medical equipment and economic status (Abdoli, 2020; Taherinia and Hassanvand, 2020). Iran is suffering from the political and economic sanctions that have directly and indirectly restricted the activities of its banking systems. This, in turn, has led to restrictions on trade, the manufacturing sector, insurance, and ventures (Abdoli, 2020). These conditions have hampered the provision of basic medical equipment for the prevention, diagnosis, and treatment of COVID-19. Concerns about the provision of equipment needed for the prevention and treatment can be one of the most important causes of fear and anxiety in the community during the COVID-19 pandemic. The COVID-19 pandemic plunged the world economy into a recession (Hashemi-Shahri et al., 2020). This recession has doubled the problems of the economy in Iran, and people are worried about unemployment, inflation, and business closures in Iran.

In this study, women presented with more symptoms of anxiety than men, and this may be related to a greater exposure of a women to stressful factors, such as a low socioeconomic status, fewer resources, lack of energy, role overload, psychological problems, and low self-esteem (Watkins et al., 2013; Carvalho et al., 2016). The lower prevalence of these symptoms among men may be attributed to what some authors have identified as men compensated differently compared with women such as the use of anger, aggressiveness, antisocial behavior, excessive consumption of alcohol, smoking, and hostility (Watkins et al., 2013; Carvalho et al., 2016). Contrary to our findings, Broche-Pérez et al. in Cuba showed that anxiety did not differ between genders (Broche-Pérez et al., 2021).

The WHO considers the COVID-19 pandemic to be a stressful and anxious time for people (World Health Organization, 2021). One of the reasons for stress and anxiety during the COVID-19 pandemic is the extensive news coverage of coronavirus causing stress and anxiety. “Headline stress disorder” was first coined by Dr. Steven Stosny who referred to mental disorders such as stress and anxiety being caused by excessive attention to news coverage. Also, the use of mobile phones provides wide news coverage (Dong and Zheng, 2020). Until 2018, Iran had an estimated Internet penetration rate of between 64 and 69% out of a population of about 82 million, about 56,700,000, that increased recently (Wikipedia, 2020). This study is limited to internet users, which include about 68% of the population of Iran.

Connor and Davidson (2003) describe resilience as an ability to cope with stress. Consistent with the present study, the average psychological resilience score of the hospital staff after the outbreak of the respiratory syndrome in South Korea showed good resilience (Son et al., 2019). In another study, most of the employees in Sierra Leone (in West Africa) had a resilience score of 71–80 during the Ebola epidemic (Colorado, 2017), indicating a high resilience; our results are similar. Similarly, Bonnano (2004) defined resilience as the ability of an individual to maintain a stable psychological equilibrium; this is the counterpart to psychological vulnerability. According to these definitions, resilience differs from recovery, accounting not for the ability of an individual to “bounce back” after a negative experience but for the ability of an individual maintain a steady psychological state despite the changing circumstances (Seery, 2011).

Despite the long-term sanctions on Iran, the people have faced and struggled with many problems (Abdoli, 2020). With their minimum facilities and maximum capabilities, they have used the opportunities for progress (Agheli and Emamgholipour, 2020). This long-term compatibility is probably one of the reasons for the high resilience of the Iranian people.

The presence or absence of resilience greatly affects the response of an individual to adverse life events. Individuals with low resilience are more likely to experience psychological distress following an adverse life event than individuals who report high resilience (Faircloth, 2017). Differences in resilience accounted for a variation in emotional responses following adverse experiences. High accounts of resilience resulted in weaker associations between stressful events and the emotional state of an individual (Ong et al., 2006). The relationship between a high level of resilience and men in the present study may be because women use coping strategies more frequently, while men focus on the problem itself, in which an individual opts to solve difficulties and attitudes in order to be able to deal with the habitual pressure, decreasing or even eliminating situations that generate stress (Bazrafshan et al., 2014; Carvalho et al., 2016).

The WHO has six recommendations for the mental and psychological well-being of people in a community, working together as one community, and supporting the medical staff. Also, instead of negative thoughts and excessive attention to news, the experiences of people who have recovered from the disease have to be followed up (World Health Organization, 2021).

The comprehensive support of the people from the government, for example, easy access to preventive equipment, rapid and free vaccination of the people, support of harmful businesses in the COVID-19 pandemic, and redoubled efforts to control the epidemic in Iran, can reduce the psychological pressure of the people in this pandemic.

Limitations of this study include the use of a snowball-sampling method. Given the emergence of this health crisis, this sampling method was considered to be most appropriate. Random sampling was not an option due to the lack of a sampling frame. However, the large sample size of this study that covered about one-tenth of a percent of the Iran population was a strong representation of Iranian society. The web-based data collection could however be a limitation, as not everyone in Iran has access to the web. This problem was minimized because a link to the questionnaire was published on Instagram, WhatsApp, and Telegram in order to be inclusive to the majority of the news channels of the provinces of Iran. Another limitation of this study was that the COVID-19 status of the participants was not obtained. This omission is important as psychological stress is likely to be much higher in those who were infected with COVID-19 than those who were not infected.

## Conclusions

The findings of this study showed a high-to-moderate level of anxiety and resilience and a low-to-moderate stress in this Iranian population. These findings suggest that there is a need for psychological interventions. An emphasis on increasing and continuous monitoring of mental health services in the health centers is recommended. The high and moderate levels of anxiety and stress in Iranians can negatively affect the well-being and performance of the population and can lead to serious problems. Also, a high resilience during negative life events is associated with well-being. The results of this study can be used to design psychological interventions. A focus on developing resilience skills may reduce psychological disorders against the COVID-19 pandemic.

## Data Availability Statement

The original contributions presented in the study are included in the article/supplementary material, further inquiries can be directed to the corresponding author/s.

## Ethics Statement

The studies involving human participants were reviewed and approved by IR.MAZUMS.REC.1399.7293. Written informed consent to participate in this study was provided by the participants' legal guardian/next of kin. Written informed consent was obtained from the individual(s), and minor(s)' legal guardian/next of kin, for the publication of any potentially identifiable images or data included in this article.

## Author Contributions

EA, ST, and HS contributed significantly in designing, collecting data, and writing articles. RE analyzed the data. VK, EF, and SP were involved in interpreting the findings and reviewing the manuscript. All authors were read and approved the final version.

## Funding

The research leading to these results was financed by the Mazandaran University of Medical Sciences.

## Conflict of Interest

The authors declare that the research was conducted in the absence of any commercial or financial relationships that could be construed as a potential conflict of interest.

## Publisher's Note

All claims expressed in this article are solely those of the authors and do not necessarily represent those of their affiliated organizations, or those of the publisher, the editors and the reviewers. Any product that may be evaluated in this article, or claim that may be made by its manufacturer, is not guaranteed or endorsed by the publisher.

## Abbreviations

STAI: State-Trait Anxiety Inventory
CD-RISC: Connor–Davidson Resilience Scale
IES-R: Impact of Event Scale-Revised
GEE: Generalized estimating equations
CIs: Confidence intervals
OR: Odds ratio.
